# Publisher Correction: A stretchable, mechanically robust polymer exhibiting shape-memory-assisted self-healing and clustering-triggered emission

**DOI:** 10.1038/s41467-023-44618-9

**Published:** 2024-01-04

**Authors:** Xiaoyue Wang, Jing Xu, Yaoming Zhang, Tingmei Wang, Qihua Wang, Song Li, Zenghui Yang, Xinrui Zhang

**Affiliations:** 1 Key Laboratory of Science and Technology on Wear and Protection of Materials, Lanzhou Institute of Chemical Physics, Chinese Academy of Sciences, Lanzhou, 730000 China; 2 Center of Materials Science and Optoelectronics Engineering, University of Chinese Academy of Sciences, Beijing, 100049 China; 3 State Key Laboratory of Solid Lubrication, Lanzhou Institute of Chemical Physics, Chinese Academy of Sciences, Lanzhou, 730000 China

**Keywords:** Polymers, Supramolecular polymers, Mechanical properties

Correction to: *Nature Communications* 10.1038/s41467-023-40340-8, published online 05 August 2023

In the Acknowledgements section of this article the grant number relating to the National Natural Science Foundation of China given for Song Li was incorrectly given as 5220523 and should have been 52205234. The original article has been corrected.

