# Supplementary material for: A stretchable, mechanically robust polymer exhibiting shape-memory-assisted self-healing and clustering-triggered emission
Source: Nat Commun. 2023 Aug 5;14:4712. doi: 10.1038/s41467-023-40340-8 (PMC10404225; doi:10.1038/s41467-023-40340-8)
Supplement: Supplementary file 1 — Supplementary Information [file 41467_2023_40340_MOESM1_ESM.pdf]

Supplementary Information

A stretchable, mechanically robust polymer exhibiting shape-memory-assisted self-healing and clustering-triggered emission

Xiaoyue Wang[1,2], Jing Xu [1,2], Yaoming Zhang[1], Tingmei Wang[1,2], Qihua Wang[1,2,3], Song Li[1*], Xinrui Zhang[1*], Zenghui Yang[1*]

[1]Key Laboratory of Science and Technology on Wear and Protection of Materials, Lanzhou Institute of Chemical Physics, Chinese Academy of Sciences, Lanzhou 730000, P.R. China

[2]Center of Materials Science and Optoelectronics Engineering, University of Chinese Academy of Sciences, Beijing 100049, P.R. China

[3]State Key Laboratory of Solid Lubrication, Lanzhou Institute of Chemical Physics, Chinese Academy of Sciences, Lanzhou 730000, China

[*]Corresponding Authors: Song Li: lisong@licp.cas.cn; Xinrui Zhang: xruiz@licp.cas.cn; Zenghui Yang: yangzh@licp.cas.cn

Table of Contents

1. Supplementary computational details 1

2. Supplementary Table and Figures 2

3. Supplementary references 10

1. Supplementary computational details

The geometric structures of the monomers, ligand and complexes (Supplementary Fig. 1) were optimized using M06-2X[1-4] functional with Grimme's dispersion (D3)[5] via Gaussian 09 program[6]. For C, H, N and O atoms, the 6-31G(d,p) basis set[7] was used, while for Zn atom, the Def2-TZVP basis set[8] was used. The vibration frequency calculations were also carried out at the same level of theory to obtain thermal corrections to free energy and ensure that all structures have no imaginary frequencies. The solvent effect of N,N-dimethylacetamide used in experiments was considered by employing the solvation model based on density (SMD)[9] and the temperatures were set to be 333.15 K which are in agreement with the experimental conditions. Single-point calculations for optimized structures were carried out at M06-2X-D3/Def2-TZVPP level of theory to obtain more accurate electronic energy. In all cases, the ultrafine grid was employed. For all hydrogen-bonding dimeric complexes, the interaction energies ($\Delta Eint$) were calculated considering the basis set superposition error (BSSE) correction[10], while for the metal-ligand complex, the bind energy (ΔEb) is the difference between the total energy of the complex and the sum of total energies of its components.

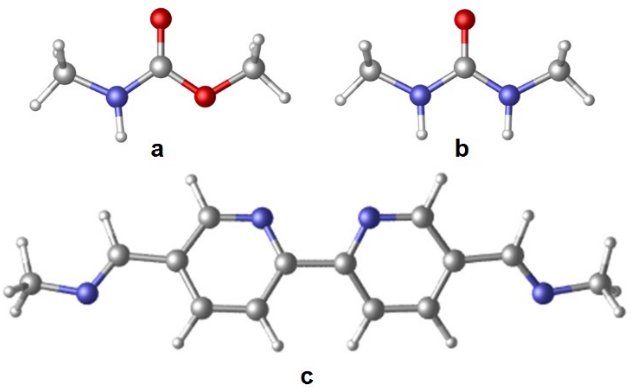

Supplementary Fig. 1 The optimized geometries of urethane (a), urea (b), and the ligand (c).

2. Supplementary Table and Figures

	HDI	PCDL	SDH	BIDI	Zn (OTf)$_2$
PUU-0	2	1	2	1	0
PUU-0.25	2	1	2	1	0.25
PUU-0.5	2	1	2	1	0.5
PUU-0.75	2	1	2	1	0.75
PUU-1	2	1	2	1	1

Supplementary Table 1 The mole ratio of the functional groups of monomers.

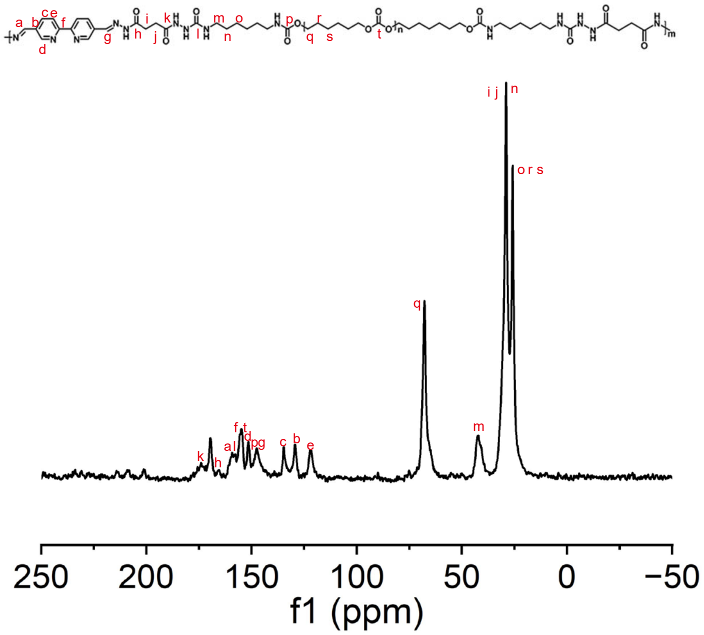

Supplementary Fig. 2 The solid state ^{13}C NMR of PUU-0.

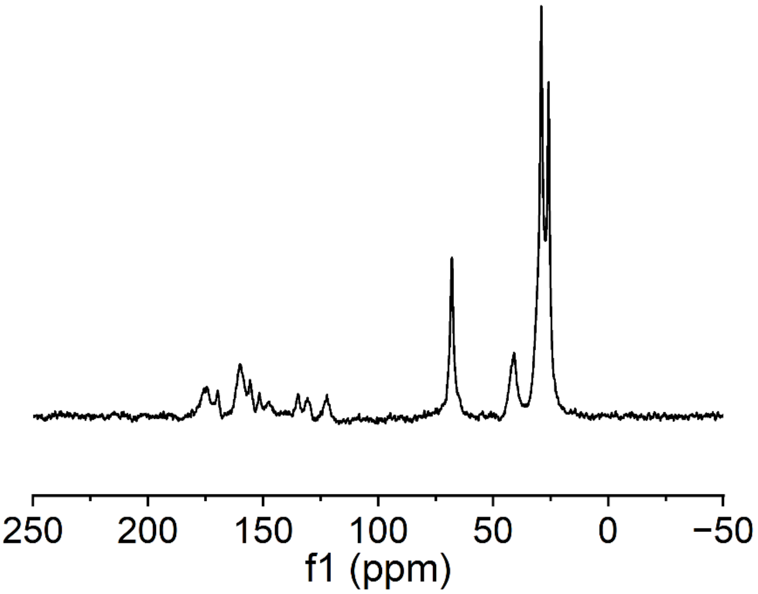

Supplementary Fig. 3 The solid state ^{13}C NMR of PUU-0.25.

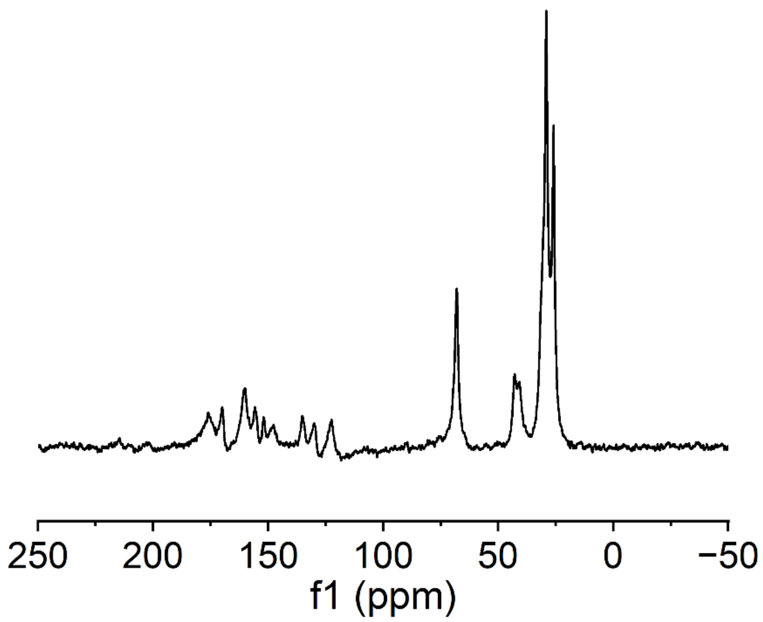

Supplementary Fig. 4 The solid state ^{13}C NMR of PUU-0.5.

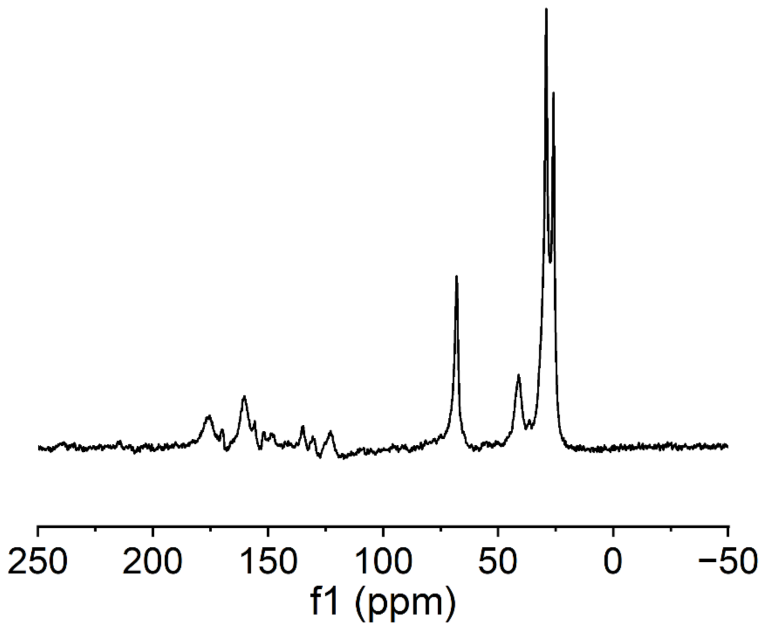

Supplementary Fig. 5 The solid state ^{13}C NMR of PUU-0.75.

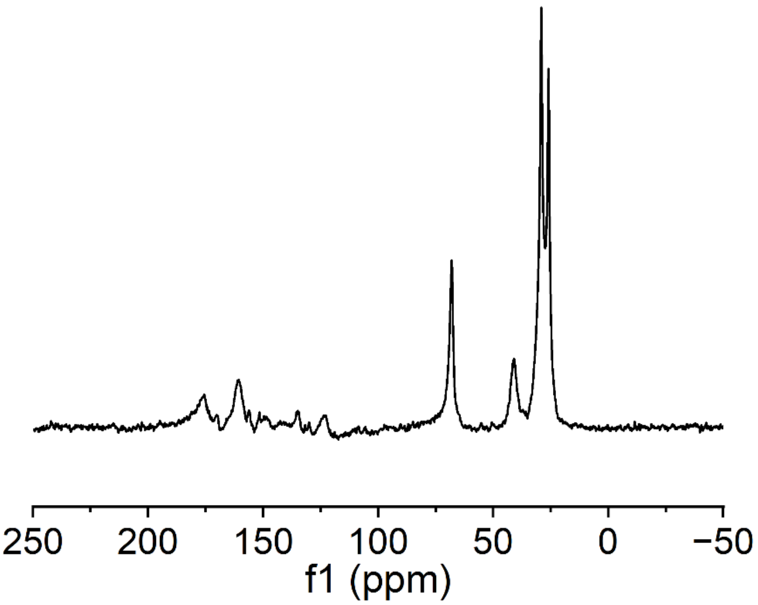

Supplementary Fig. 6 The solid state ^{13}C NMR of PUU-1.

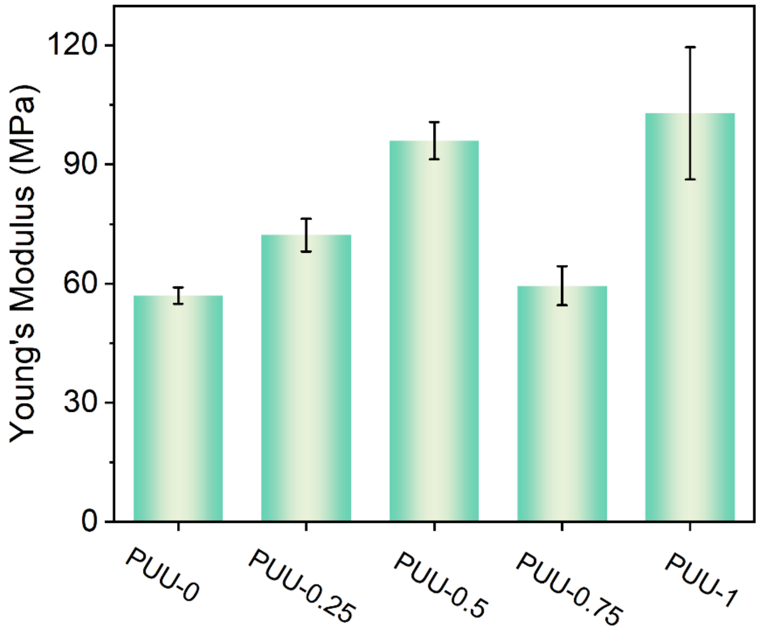

Supplementary Fig. 7 Young′ s modulus of PUUs. Shown are the mean ± standard deviation of $n = 3$ independent experimental repeats.

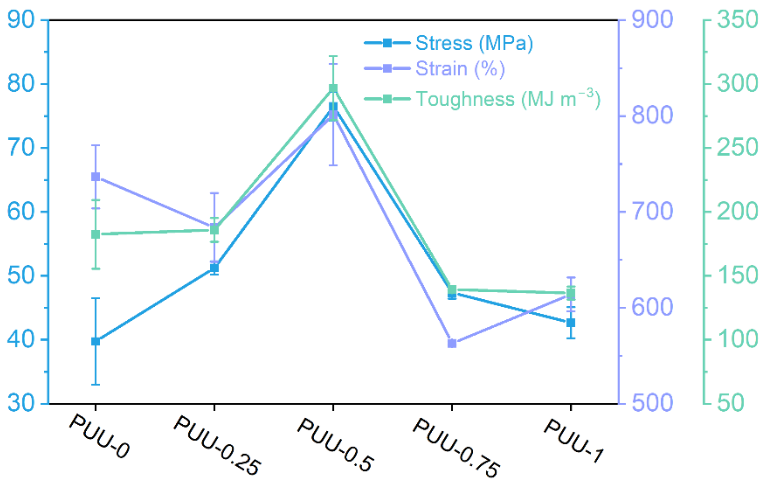

Supplementary Fig. 8 Stress, strain and toughness of PUUs. Shown are the mean ± standard deviation of $n = 3$ independent experimental repeats.

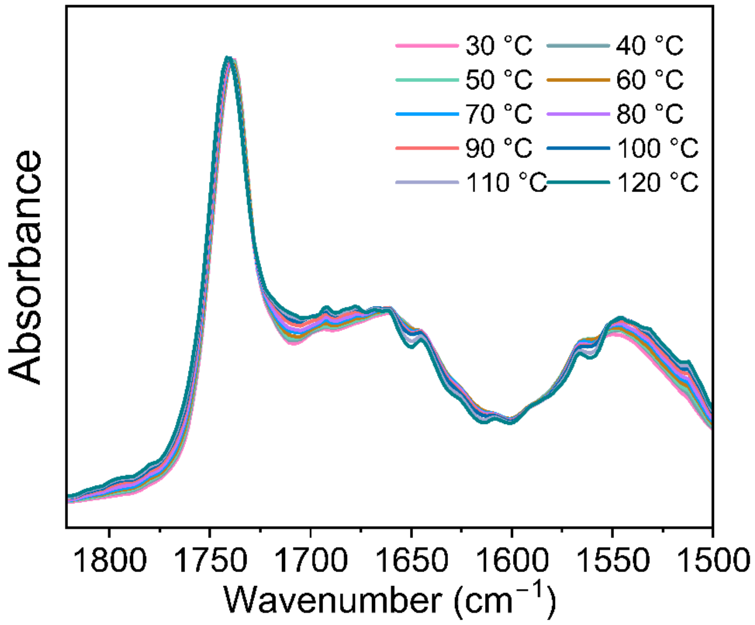

Supplementary Fig. 9 The *in-situ* temperature-dependent FTIR of PUU-0.5.

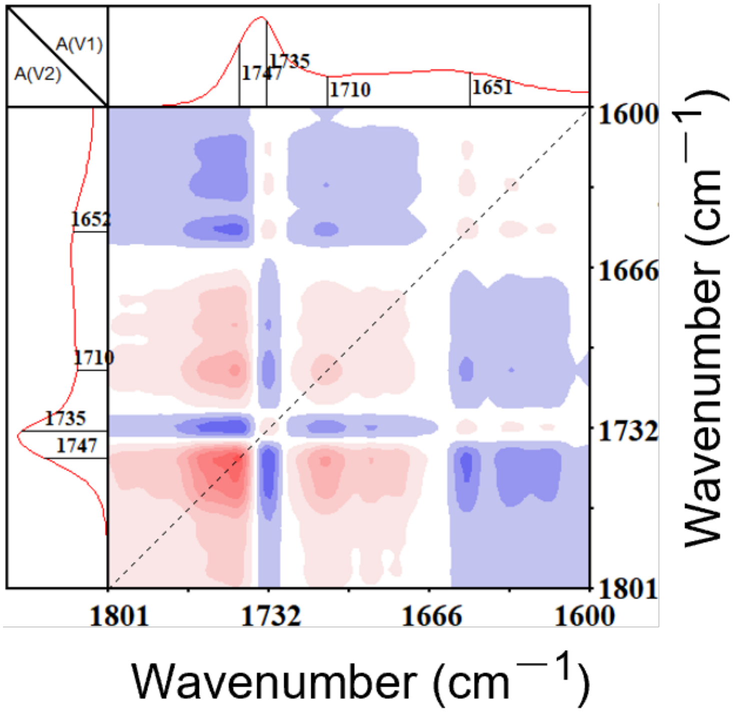

Supplementary Fig. 10 The synchronous spectrum of the PUU-0.5.

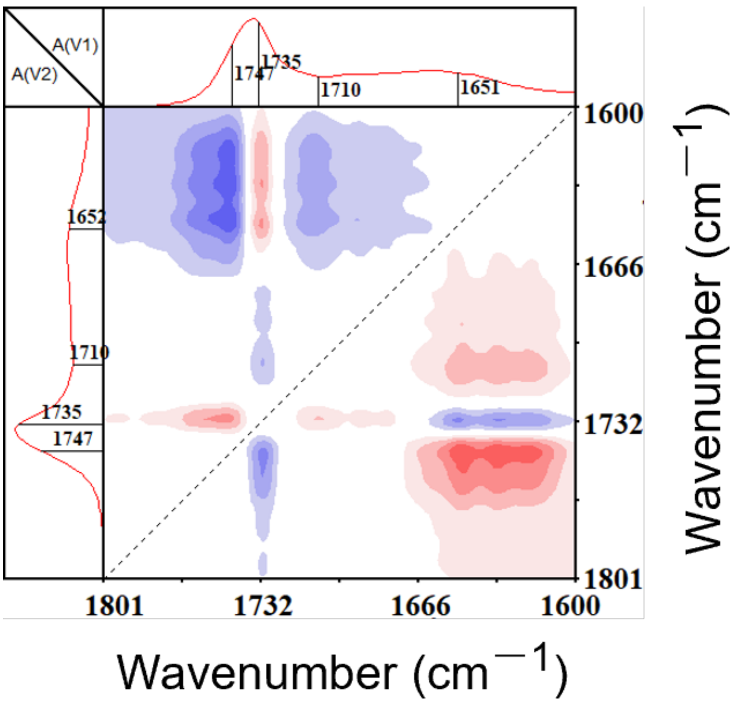

Supplementary Fig. 11 The asynchronous spectrum of the PUU-0.5.

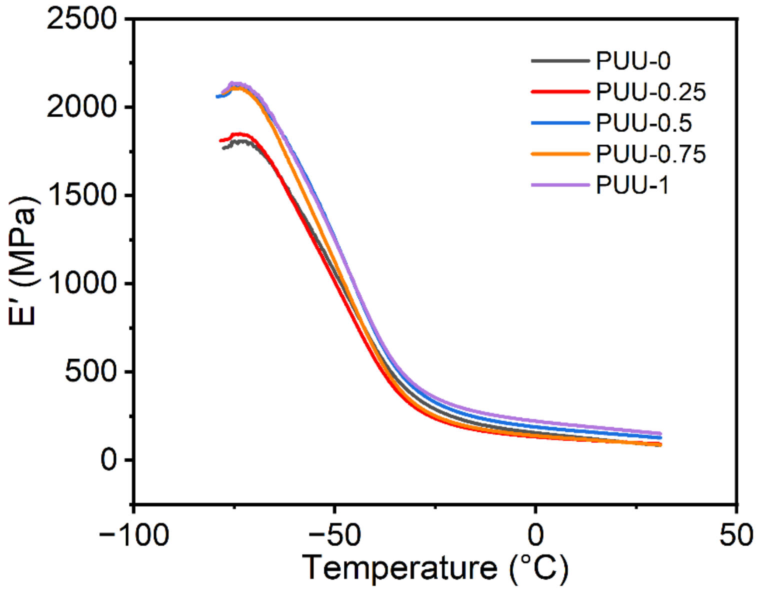

Supplementary Fig. 12 The E′ of PUUs.

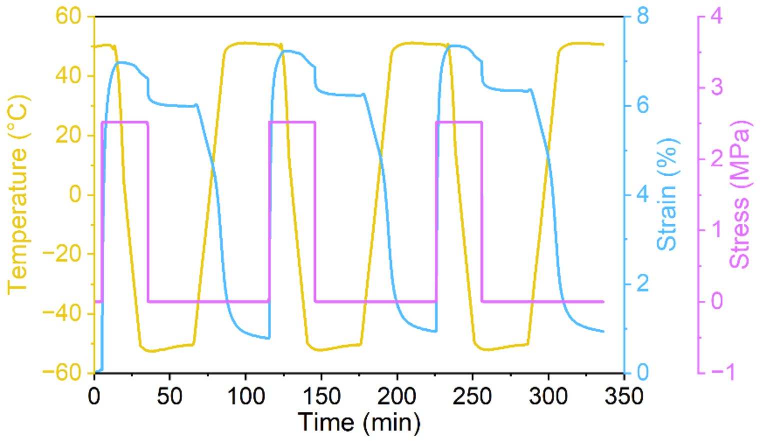

Supplementary Fig. 13 Shape memory cycle curves of PUU-0.

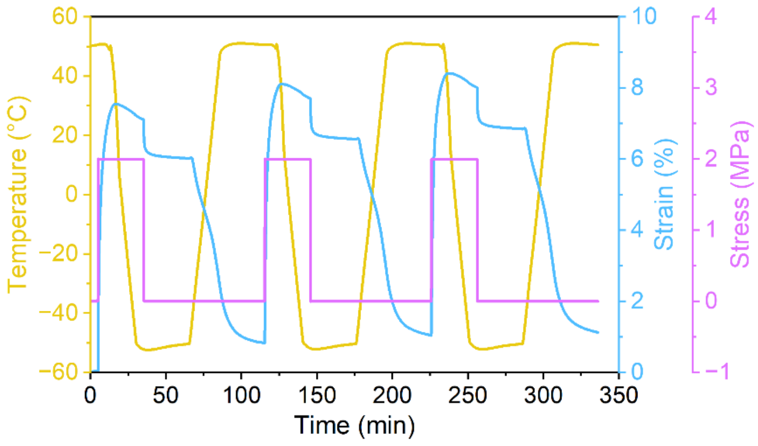

Supplementary Fig. 14 Shape memory cycle curves of PUU-0.25.

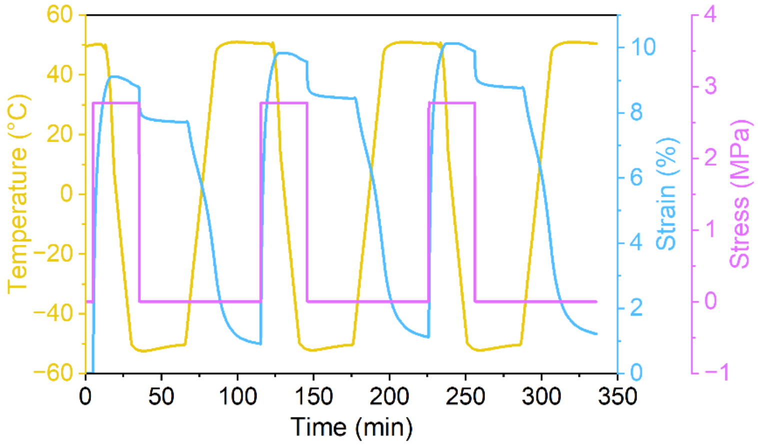

Supplementary Fig. 15 Shape memory cycle curves of PUU-0.75.

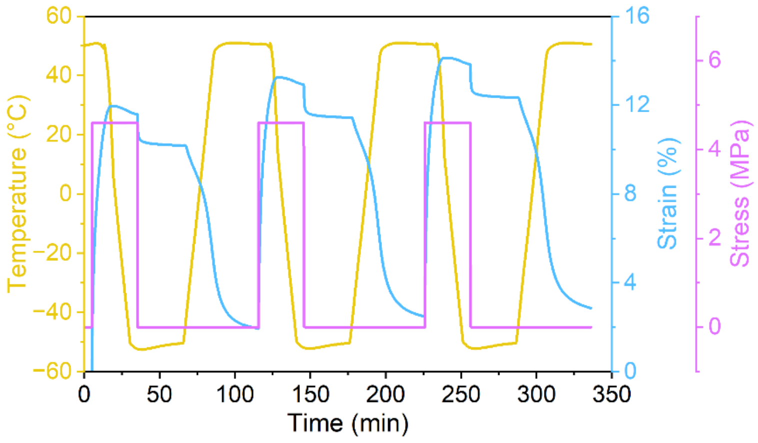

Supplementary Fig. 16 Shape memory cycle curves of PUU-1.

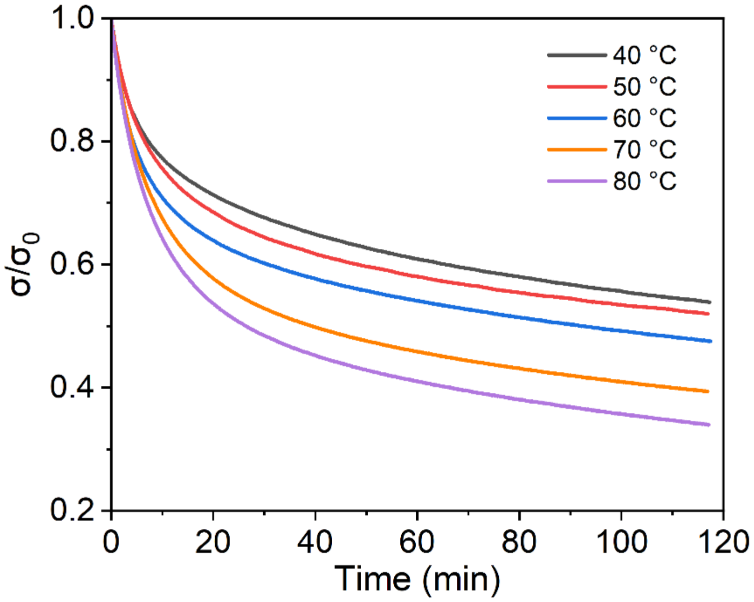

Supplementary Fig. 17 The stress-relaxation curves of PUU-0.5 at different temperature.

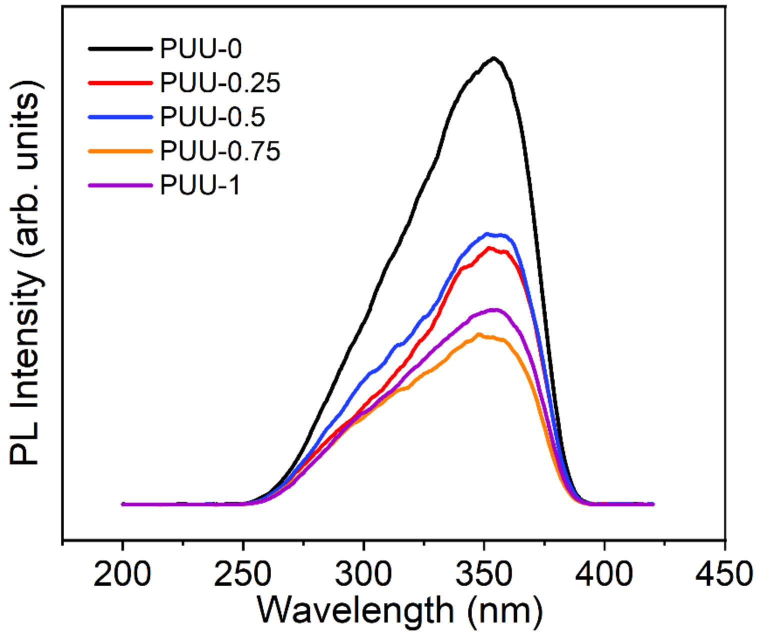

Supplementary Fig. 18 PL spectra of PUU-X.

3. Supplementary references

1. Zhao, Y. & Truhlar, D. G. The M06 suite of density functionals for main group thermochemistry, thermochemical kinetics, noncovalent interactions, excited

states, and transition elements: two new functionals and systematic testing of four M06-class functionals and 12 other functionals. *Theor. Chem. Acc.* 120, 215-241, (2008).

2. Koleva, G., Galabov, B., Kong, J., Schaefer, H. F. & Schleyer, P. v. R. Electrophilic aromatic sulfonation with SO_3: concerted or classic S_EAr mechanism? *J. Am. Chem. Soc.* 133, 19094-19101, (2011).
3. Zade, S. et al. Products and Mechanism of Acene Dimerization. A Computational Study. *J. Am. Chem. Soc.* 133, 10803-10816, (2011).
4. Breugst, M., Eschenmoser, A. & Houk, K. Theoretical Exploration of the Mechanism of Riboflavin Formation from 6,7-Dimethyl-8-ribityl-lumazine: Hydride Transfer, Hydrogen Atom Transfer, Nucleophilic Addition, or Nucleophilic Catalysis? *J. Am. Chem. Soc.* 135, 6658-6668, (2013).
5. Grimme, S., Antony, J., Ehrlich, S. & Krieg, H. A consistent and accurate ab initio parametrization of density functional dispersion correction (DFT-D) for the 94 elements H-Pu. *J. Chem. Phys.* 132, 154104-154123, (2010).
6. M. J.Frisch, et.al. Gaussian 09, Revision D.01, Gaussian, Inc., Wallingford, CT, (2009).
7. Spitznagel, G. W., Clark, T., von Ragué Schleyer, P. & Hehre, W. J. An evaluation of the performance of diffuse function-augmented basis sets for second row elements, Na-Cl. *J. Comput. Chem.* 8, 1109-1116, (1987).
8. Schäfer, A., Huber, C. & Ahlrichs, R. Fully optimized contracted Gaussian basis sets of triple zeta valence quality for atoms Li to Kr. *J. Chem. Phys.* 100, 5829-

5835, (1994).

9. Marenich, A. V., Cramer, C. J. & Truhlar, D. G. Performance of SM6, SM8, and SMD on the SAMPL1 Test Set for the Prediction of Small-Molecule Solvation Free Energies. *J. Phys. Chem. B* 113, 4538-4543, (2009).

10. Boys, S. F. & Bernardi, F. The calculation of small molecular interactions by the differences of separate total energies. Some procedures with reduced errors. *Mol. Phys.* 19, 553-566, (1970).
